# Prediction of Temperature Distribution in Concrete under Variable Environmental Factors through a Three-Dimensional Heat Transfer Model

**DOI:** 10.3390/ma15041510

**Published:** 2022-02-17

**Authors:** Haoyu Zeng, Chao Lu, Li Zhang, Tianran Yang, Ming Jin, Yuefeng Ma, Jiaping Liu

**Affiliations:** 1 School of Material Science and Engineering, Southeast University, Nanjing 211189, China; 220202234@seu.edu.cn; 2 Division of Science and Technology Management, China Three Gorges Corporation, Wuhan 430010, China; lu_chao1@ctg.com.cn (C.L.); zhang_li6@ctg.com.cn (L.Z.); yang_tianran@ctg.com.cn (T.Y.); 3 College of Materials Science and Engineering, Chongqing University, Chongqing 400045, China; myf@cqu.edu.cn

**Keywords:** concrete, temperature distribution, natural environment, heat transfer, FE coupling model

## Abstract

Temperature distribution in concrete is significant to the concrete structure’s macro properties and different factors affect the heat transfer in concrete, and therefore influence the temperature distribution. This work established a three-dimensional transient heat transfer model coupled with various environmental factors, using the finite element method for calculating the results and real-measured data for testing accuracy. In addition, a sensitivity evaluation of various factors was conducted. Due to various environmental factors, the results revealed that the prediction of temperature distribution in concrete by the three-dimensional model had great accuracy with an error of less than 4%. A particular hysteresis effect of temperature response in the concrete existed. Considering heat transfer in different spatial directions, the model can predict the temperature change of each spatial point instead of the spatial surface in different depths, proving the shortcomings of a one-dimensional heat transfer model. A greater solar radiation intensity caused a more significant temperature difference on the concrete surface: the surface temperature difference in July was twice as significant as that in December. Wind speed had a cooling effect on the concrete surface, and stronger wind speed accompanied with a stronger cooling effect made the surface temperature closer to the ambient temperature. Material properties had different effects on the temperature distribution of the surface part and internal part: the specific heat capacity determined the speed of the outer layer temperature change while the thermal conductivity determined the speed of the inner layer temperature change.

## 1. Introduction

Most concrete structures are located in the atmosphere, suffering from environmental conditions such as sunshine, wind, and changing temperature. The surface temperature of concrete buildings changes immediately with the alternation of external radiation, convection, and ambient temperature. Due to the concrete surface being directly exposed to atmospheric factors, it has a quicker response to the external temperature than internal concrete, resulting in inhomogeneous internal temperature distribution, that is, temperature field. On the one hand, temperature distribution directly leads to deformations of concrete such as thermal load [1], expansion [2], contraction [3], and bending [4], which probably influence the original integrity of concrete structures. On the other hand, the temperature field indirectly produces impacts on the internal micro environment [5] and results in durability issues such as carbonation [6], chloride penetration [7,8,9], steel bar corrosion [10,11], and creep [12] of the concrete structures, which consequently induce the degradation of long-term durability of concrete. For instance, the chloride corrosion rate doubles with a 10 °C increase [13], steel bar corrosion rates exhibit significant differences under different curing temperatures [14], and a linear correlation exists between carbonation depth and temperature [6]. Therefore, the prediction of the temperature distribution offers a necessary and practical experimental value in the life-cycle service assessment of concrete. Furthermore, previous studies on inner temperature prediction have been gradually applied for optimizing the thickness of building insulation materials and ascertaining the compatibility of multi-layer materials (including adhesives and coatings on building surfaces), which can significantly improve the rationality and functionality of the architectural design.

Theories from different aspects have been put forward to predict the change of temperature field in concrete structures under the natural environment. From the perspective of fluctuating ambient temperature, Luikov [15] applied the analytical method and the one-dimensional transient heat transfer equations so as to ascertain the temperature field by assuming the ambient temperature fluctuating in the form of a simple harmonic. Campo [16] proposed one-dimensional analytical solutions of temperature distribution equations for different shapes of specimens based on the nomograph theory, and realized the internal temperature prediction. Yuan et al. [17] developed a one-dimensional temperature predicting model using the extreme difference dissection method.

With an in-depth understanding of the actual natural conditions, radiation and atmospheric convection were investigated to affect the inner temperature distribution, and were then incorporated into prediction models experimentally and theoretically. For convection, Saetta et al. [18] obtained a relational formula between convective coefficient and wind velocity through a numerical method. Zhang et al. [19] carried out a wind tunnel test of concrete and drew a relationship between convection heat transfer and wind velocity. In terms of radiation, Berdah [20] used a spectral radiometer to monitor the radiation of the sky, which then drew the relationship between sky radiance and dew temperature. Xiao et al. [21] analyzed how meteorological parameters impact the temperature field of concrete structures under sunshine. Duffie et al. [22] summarized a time-varying law of radiation and its relationship with heat transfer on the material surface.

With complex boundary conditions being input, the traditional analysis methods cannot meet the requirement of accurate prediction, such that a number of numerical solutions have gradually emerged. Schindler et al. [23] adopted a high performance concrete paving (HIPERPAV) model and transient one-dimensional finite difference model to explore the temperature response of Portland cement concrete pavement under the coupled actions of external environment factors and internal heat produced by cement hydration. Cho et al. [24] established an internal temperature and relative humidity (RH) prediction model to describe the evolution of internal temperature and relative humidity in concrete under the coupled actions of radiation, convection, and ambient temperature, based on the basic principle of one-dimensional unsteady heat conduction. Likewise, Benkhaled et. al. proposed a one-dimensional FE model for predicting heat and moisture transfer through a hemp−concrete wall [25]. Qian et al. [26] developed a three-dimensional finite element model to predict the temperature distribution of an asphalt mixture during compaction based on Fourier’s law and thermodynamic principle.

However, in civil buildings, plate-type concrete structures such as walls and floors, widely assumed as infinite plates, major relevant models follow the rule of one-dimensional heat conduction. However, in the actual heat transfer process, any spatial point or plane is not isolated or linear-correlated, but interacts and cooperates under environmental conditions, which is actually a three-dimensional process. Moreover, for roads and bridges, investigations on the prediction model of temperature distribution based on three-dimensional transient heat conduction have been carried out [26,27], which serve as a reference.

This work focuses on the change of temperature distribution in concrete under a natural environment following Fourier’s law, thermodynamic principle, and numerical solution theories. First, this study applied the COMSOL finite element calculation by considering the fluctuating ambient temperature, convection, and radiation. Then, a comparison between the calculation results and the real-measured data derived from the literature [24] was made to verify the model. Consequently, a multi-factor coupling prediction model of internal temperature distribution in concrete was established and was proven to be reliable, and could provide a relatively accurate prediction of the temperature field under a natural environment. In addition, the sensitivity of the model was evaluated with respect to environmental conditions and materials properties. The interrelation between each parameter and the temperature distribution was analyzed. Practically, the model can be accessed for evaluation of the temperature change, as well as be combined with the follow-up deterioration prediction model to consider issues such as temperature stress, chloride penetration, fatigue degradation, carbonation, and other durability problems.

## 2. Temperature Response Model in Concrete Structures

### 2.1. Basic Principle

**Figure 1:**
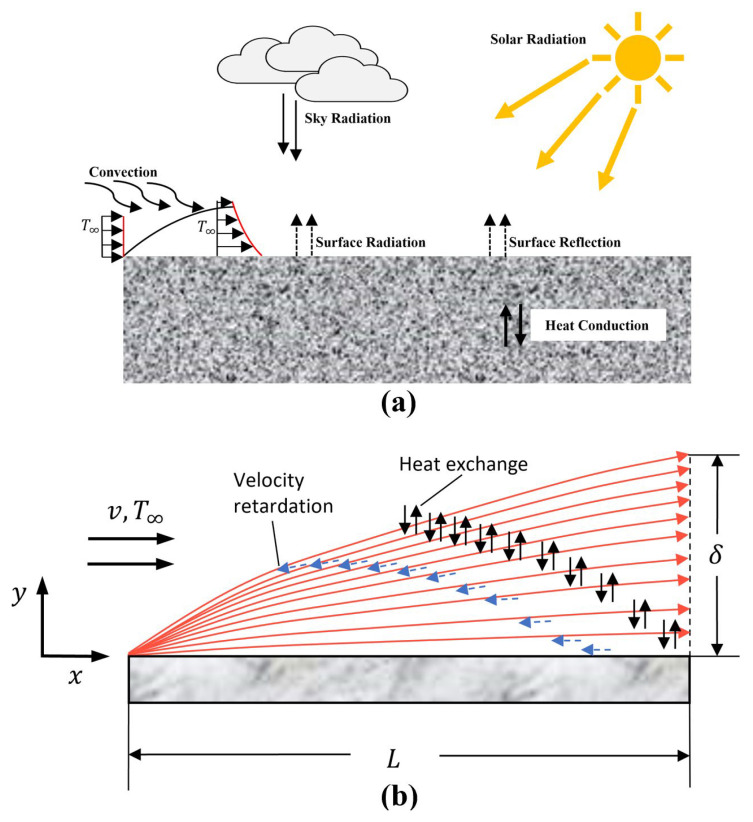
(**a**) A schematic diagram of heat transfer model of concrete exposed to various natural environmental factors and (**b**) a schematic diagram of the convective heat transfer.

As shown in Figure 1a, the upper surface of concrete exposed to the atmosphere suffers from multi-environmental factors, including atmospheric convection, radiation, and a fluctuating ambient temperature. These factors affect the heat exchange between the internal and the external environment, leading to uneven temperature distribution in concrete. 

Convective and radiant heat transfer are two primary forms occurring on the surface of the concrete. Basic principles were introduced as follows [28]:

First, in terms of convection, as shown in Figure 1b, an air layer exists between the surface and the atmosphere, where the wind speed gradient and temperature gradient cannot be ignored. Therefore, the impact of convection on the concrete structure is generally affected by wind velocity and temperature difference. Different wind speeds lead to different air layer thicknesses and thermal transfer rates, so the internal and external temperature exchange speeds differ. Different temperature differences also change the air layer thicknesses and have specific effects on surface heat transfer. Therefore, the combined effect of wind and temperature gradient, namely convection, has a significant effect on the internal temperature distribution.

Then, theoretically, all the objects in nature with the temperature above absolute zero can emit thermal radiation. In this study, radiations emitted from the sun and sky were taken into consideration. For one thing, when concrete is exposed to sunlight, only part of the radiation is absorbed by the concrete surface, but the residual radiation energy is reflected. The intensity of solar radiation is related to the time, solar altitude angle, and total solar radiation [29]. For another, sky radiation (sky irradiance) occurs between the ground and the sky. The radiation energy participates in the energy exchange with the concrete, which is of great significance in heat conduction. 

In this study, concrete was assumed as an isothermal body initially. The heat transfer between the concrete surface and surrounding environment led to differences in the temperatures of the concrete surface and internal concrete. Then, internal temperature gradients drove energy transport, which incurred the process of conduction. Consequently, the internal and external energy began to flow continuously, with the internal temperature distribution changing accordingly.

### 2.2. Governing Equation of Heat Transfer in Concrete Structure

The heat conduction inside the concrete produced by the temperature gradient follows Fourier’s law and energy conservation law [30], considering the heat conduction per unit time through a given cross section is proportional to the temperature change rate and cross section area in the perpendicular direction. The differential equation of heat transfer is established as follows, neglecting the inner heat source of materials

(1) $${\rho C}_{V}\frac{\partial T}{\partial t}=\lambda \left(\frac{\partial^{2}T}{\partial x^{2}}+\frac{\partial^{2}T}{\partial y^{2}}+\frac{\partial^{2}T}{\partial z^{2}}\right)$$

where C_V_ refers to the specific heat of the heat conductor; T refers to the inside temperature; t refers to time, ρ refers to density; λ refers to thermal conductivity; and x, y, and z refer to different direction vectors in the coordinate system. In nonlinear transient heat conduction, the left-hand side of Equation (1) denotes the time-varying item; correspondingly, the right denotes the diffusing item. 

**Figure 2:**
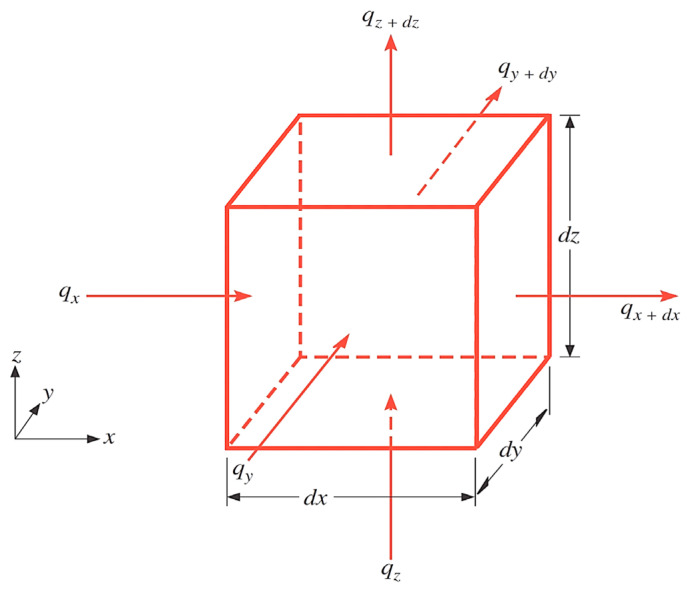
Heat conduction in differential control volume.

As shown in Figure 2, the processes of thermal conduction are described using a differential control volume with importing rate equations. 

Given a homogeneous and continuous concrete, T(x,y,z) in cartesian coordinates denotes the internal temperature of the spatial points. An infinitesimal volume element dV=dx · dy · dz is defined according to the energy conservation, as shown in Figure 2. Specifically, when a temperature gradient exists, it prompts heat conduction through each direction of the control volume. Given the energy input from one side, the opposite side draws the energy.

(2) $$q_{x+\mathrm{dx}}{=q}_{x}+\frac{\partial q_{x}}{\partial x}{;\;q}_{y+\mathrm{dy}}{=q}_{y}+\frac{\partial q_{y}}{\partial y}{;\;q}_{z+\mathrm{dz}}{=q}_{z}+\frac{\partial q_{z}}{\partial z}$$

Meanwhile, the heat flow in each direction is proportional to the corresponding temperature gradient.

(3) $$q_{x}=-\lambda \frac{\partial q_{x}}{\partial x}{;\;q}_{y}=-\lambda \frac{\partial q_{y}}{\partial y}{;\;q}_{z}=-\lambda \frac{\partial q_{z}}{\partial z}$$

Substituting Equation (3) into Equation (2), the net heat flow change through the x, y, and z directions gives the following.

### 2.3. Initial Condition and Boundary Conditions

In order to solve Equation (1), concrete structures are assumed to be isothermal at the initial moment, as in Equation (4):

(4) $$T\left(x,y,t\right){|}_{t=0}{=t}_{0\;}$$

Following energy conservation, the boundary conditions are established as Equation (5), where the left-hand side refers to the external energy, while the right refers to the internal heat conduction.

(5) $$\begin{array}{c} q_{{\mathrm{conv}}_{x}}{+q}_{{\mathrm{rad}}_{x}}=-\lambda \frac{\partial T}{\partial x}\\ q_{{\mathrm{conv}}_{y}}{+q}_{{\mathrm{rad}}_{y}}=-\;\lambda \frac{\partial T}{\partial y}\\ q_{{\mathrm{conv}}_{z}}{+q}_{{\mathrm{rad}}_{z}}=-\lambda \frac{\partial T}{\partial z} \end{array}$$

where q_cov_ stands for convective heat transfer; q_rad_ is radiation heat transfer; and the subscripts x, y, and z refer to the directions of the heat flow. 

#### 2.3.1. Convection

The convective heat transfer equation following Newton’s law of cooling [31] is calculated as follows:

(6) $$q_{\mathrm{conv}}{=h}_{c}\left(T_{s}-T_{\infty }\right)$$

where h_c_ stands for convective heat transfer coefficient, W/(m^2^·°C). 

The convective heat transfer coefficient h_c_ is related to a number of factors, such as wind speed, surface roughness, and the geometric configuration of the exposed concrete structure [32]. When the concrete is exposed to the natural environment, the expansion of the thermal boundary layer of the concrete is free of constraint, and the interaction between the fluid and the surface is driven by natural wind, which can be simplified as forced convection. In this process, the temperature and velocity gradients of the atmospheric wind zone beyond the boundary layer can be neglected.

To simplify the calculation, in the field of civil engineering, the convective heat transfer coefficient h_c_ is usually calculated by empirical formula [18], which gives the following:

(7) $$h_{c}=\{\begin{array}{c} 4.0v+5.6\;\;\;v\;\leq \;5\\ {7.15v}^{0.78}\;\;\;v\;>\;5 \end{array}$$

#### 2.3.2. Radiation

In this study, total radiation consisted of atmospheric radiation, radiation energy emitted from the surface, and solar radiation [33]. The first two terms are defined as sky radiation, as shown in Equation (8).

(8) $$q_{\mathrm{rad}}{=q}_{\mathrm{sol}}{+q}_{\mathrm{sky}}$$

where $q_{\mathrm{sol}}$ and $q_{\mathrm{sky}}$ represent solar radiation and sky radiation, respectively. 

The sky radiation absorbed by the surface of concrete is written by Equation (9):

(9) $$q_{\mathrm{sky}}=\varepsilon \sigma (T_{s}^{4}-T_{\mathrm{sky}}^{4})$$

where ε is the emissivity of a horizontal grey-body radiating surface, σ is the Stefan–Boltzmann constant, $\sigma ={5.67\;\times \;10}^{-8}W/{(m}^{2}\cdot K^{4})$, T_s_ is the absolute temperature (K) of the surface, and $T_{\mathrm{sky}}$ is sky temperature related to the weather and season; that is, clouds will make the sky temperature increase, which is higher on cloudy days than that sunny days, and the difference in atmospheric temperature and sky temperature can increase to 30 °C in winter, but decrease to 5 °C in summer [22], as shown in Equation (10):

(10) $$T_{\mathrm{sky}}{=T}_{\infty }{\left[0.711+{0.0056T}_{\mathrm{dp}}+{0.000073T}_{\mathrm{dp}\;}^{2}+0.013\cos\left(15\;t\right)\right]}^{\frac{1}{4}}$$

where $T_{\mathrm{dp}}$ is the dew point temperature (°C). 

Solar radiation is a variation related to solar altitude angle, time, and total solar radiation. As mentioned above, partial sunlight reaching the surface of concrete is absorbed by the concrete, which is expressed as [34]:

(11) $$q_{\mathrm{sol}}(t)=\alpha \{\begin{array}{cc} 0, & [0,\frac{\pi }{\omega }\left(1-\frac{m}{2}\right)],\\ q_{0}\cos[m\omega (t-12)], & [\frac{\pi }{\omega }\left(1-\frac{m}{2}\right),\frac{\pi }{\omega }\left(1+\frac{m}{2}\right)],\\ 0, & [\frac{\pi }{\omega }\left(1+\frac{m}{2}\right),\frac{2\pi }{\omega }], \end{array}$$

where $q_{0}$ stands for maximum solar radiation at 12:00 a.m., $q_{0}=0.131{\mathrm{mq}}_{d}$; $q_{d}$ represents the total solar radiation reaching the atmosphere; m=12/C(t), C(t) is the duration of sunlight in one day, ω=2π/24; and α is absorptivity, which is 0.65 for concrete [18].

### 2.4. Finite Element Method (FEM)

To solve the nonlinear differential equations with complex, analytic methods following the theories of separation of variables [35], finite integral transforms [36,37,38], and Laplace transform [39] were put forward. However, the limitations resulting from complex boundary conditions made the analytic methods inapplicable when the numerical methods emerged. There are two practical solving methods: finite element method (FEM) and finite difference method (FDM). In this study, we applied FEM to obtain the approximate solution.

## 3. Verification

### 3.1. Selection of the System

**Figure 3:**
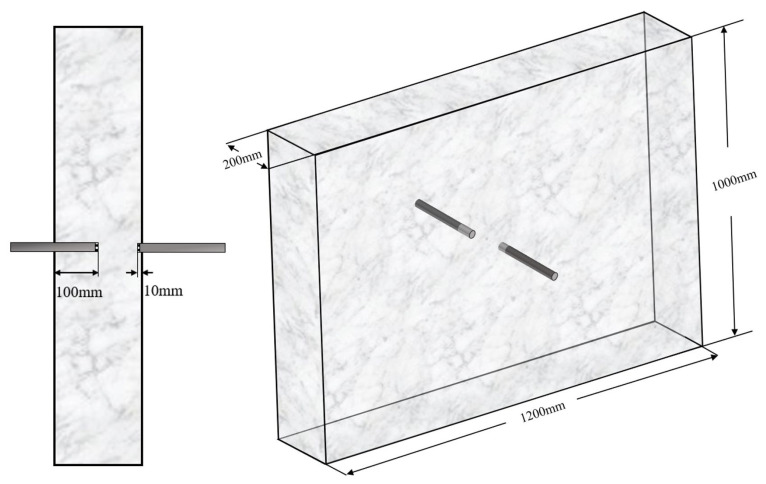
Concrete specimen and setup for temperature sensors [24].

To verify the reliability and accuracy of this numerical analysis model, a comparison was conducted between the simulated results and the experimental results of ordinary Portland concrete from the literature [24]. The mixture proportions of concrete are listed in Table 1. Concrete specimens with dimensions of ${\;1000\;\times \;1200\;\times \;200\;\mathrm{mm}}^{3}$ were made. As shown in Figure 3, temperature−humidity sensors were embedded at a distance of 500 mm from the upper side, 600 mm from the lateral sides, and 190/100 mm from the opposite side. To make the initial temperature uniform, concrete specimens were cured under standard conditions for 28 days and under a sealed chamber for 14 days, and then directly exposed to the natural atmosphere of Tsukuba City (Japan) for 100 days. According to the meteorological data presented in the literature [24], the climate conditions during the testing period were introduced as below: the average temperature was 16.1 °C with the highest temperature being 37.1 °C and the lowest temperature being −5.0 °C; the average wind velocity was 2.4 m/s, and the maximum solar radiation was 3.6 MJ/m^2^. Furthermore, as listed in Table 2, concrete and boundary parameters such as thermal conductivity (Khan [40]), sky temperature (Duffie [22]), and specific heat capacity (Choktaweekarn [41]) were incorporated into the multifactor-coupled numerical simulation to improve the accuracy of the prediction model.

**Figure 4:**
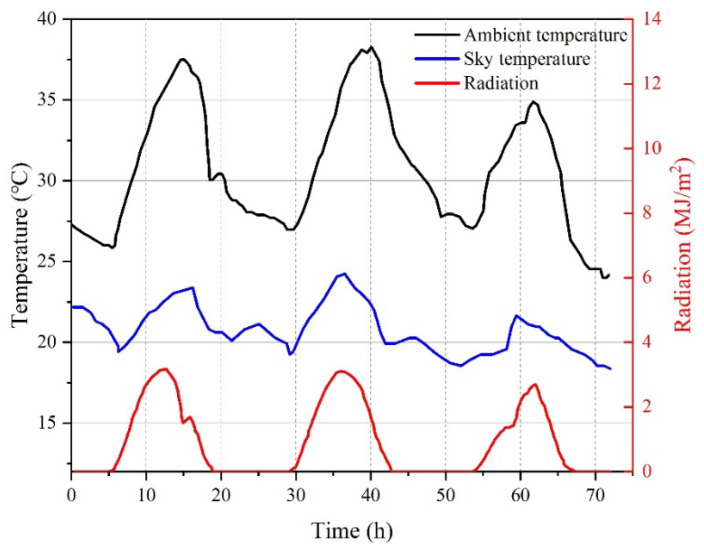
Real-measured ambient temperature and solar radiation curve (15–18 August 2007) [24].

The input of parameters such as ambient temperature, sky temperature, and amount of solar radiation was based on real-detected data, as shown in Figure 4. The temperature range was between 25.9 °C and 37.5 °C, with a mean temperature of 32 °C. The solar radiation lasted from 06:00 a.m. to 18:00 p.m., with a maximum radiation intensity of about 2.5 MJ/m^2^ at noon.

### 3.2. Accuracy Analysis

**Figure 5:**
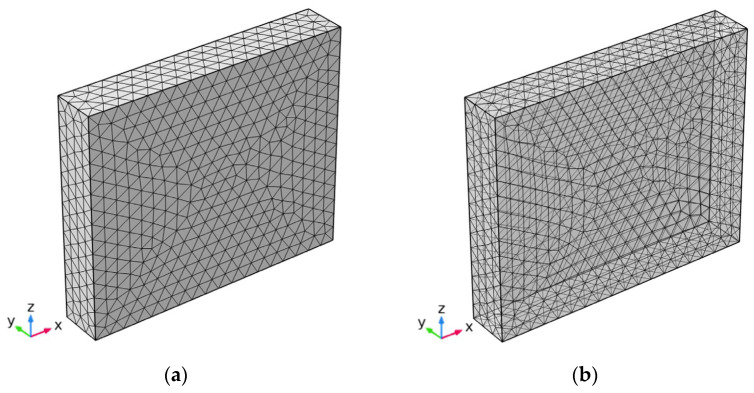
Construction of the finite element mesh model. (**a**) Stereogram; (**b**) Perspective.

The finite element method was conducted through COMSOL Multiphysics^®^ (Fluid Flow & Heat Transfer Modules, COMSOL Inc., Stockholm, Sweden). The physical fields interface for SOLID HEAT TRANSFER and SURFACE TO SURFACE RADIANT HEAT TRANSFER were incorporated into the coupled analysis by inputting the parameters of materials properties and boundary conditions. In the three-dimensional model, *x*, *y*, and *z* axes represented the width, length, and height, respectively. The six flat concrete surfaces were divided into 1848 triangular boundary elements and the internal domain was divided into 12,355 tetrahedron domain elements, as shown in Figure 5. Different concrete surfaces were implemented with different environmental conditions based on the actual situations. Specifically, forced convection acted on each surface except the bottom, as did solar radiation. It was assumed that the initial temperature of the model was the same as the actual initial temperature. Ambient temperature, speed velocity, and solar radiation were in accordance with local weather and the literature [24]. 

**Figure 6:**
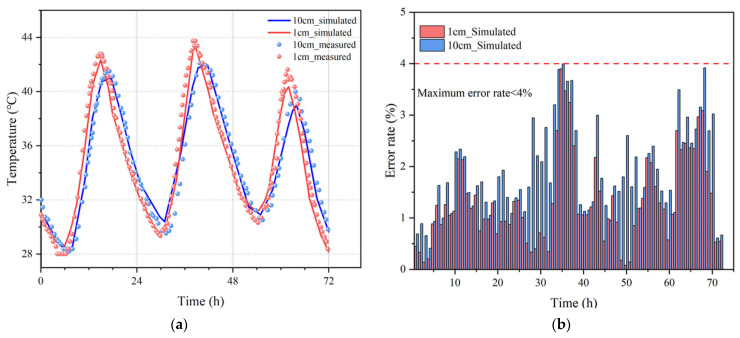
Comparison of measured data and simulated data with error analysis. (**a**) Comparison between the measured and simulated data; (**b**) Error analysis of different depths.

Figure 6 shows the real-measured and simulated temperature data at different depths in the concrete within three days (the initial temperature of concrete was set to be 31 °C). The simulated temperatures were in good accordance with the real-measured temperatures, especially in the peaks and valleys of the curves, where the simulated curve could relatively envelop the measured data. Although, due to the iterative calculation from outside to inside, the accumulation of the truncation error was propagated layer by layer, which incurred the accuracy of the model on the wane as testing depth moved forward. However, the differences (errors) between the real-measured and simulated temperatures were shown to be less than 4% within each hour. The above analysis proves the reliability and accuracy of our proposed model with consideration of multi-dimension and multiple boundary conditions. This predicted model could precisely predict the internal temperature distribution at different periods in a natural environment with convection and radiation.

**Figure 7:**
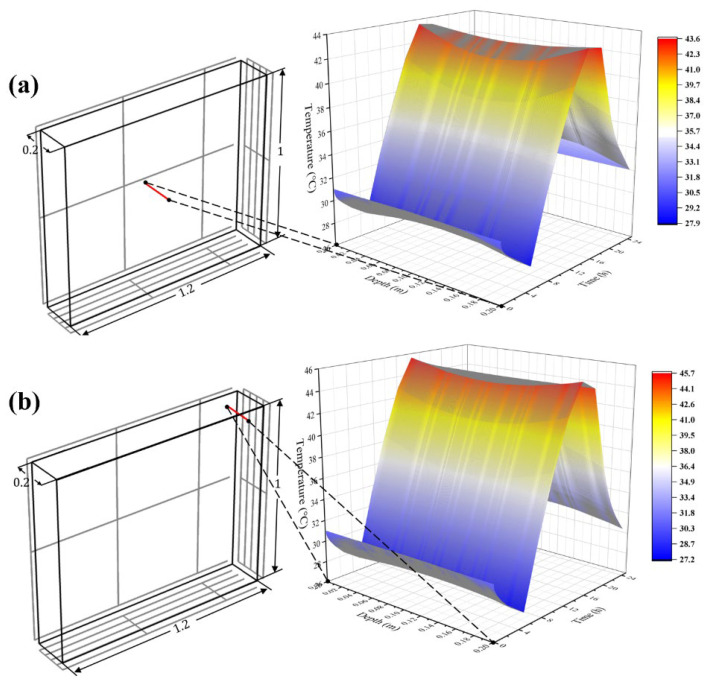
Cloud diagram of concrete internal temperature distribution based on the time-varying model: (**a**) center line; (**b**) corner line.

To ascertain the impact of dimension on the model, it was assumed that the specimens were similar to concrete walls. As shown in Figure 7, the red line denotes all points along the thickness direction at different locations. Specifically, the red line in Figure 7a was in the middle of the wall, where the sensors were embedded. The red line in Figure 7b was in the corner, which was relatively near to the environmental conditions.

Due to direct exposure to the natural environment, the temperature of vertical-side surfaces tended to change more quickly; the inside more slowly. The peak of the contour displayed an apparent “∪” or “∩” shape, which verified that there was a delay in heat transfer.

In Figure 7a, in contrast to Figure 7b, due to the location in the center of the concrete, the points in the red line suffered from relatively weaker environmental conditions, that is, the change frequency of three-dimensional heat conduction was lower. This means a stronger hysteresis effect and weaker temperature fluctuation existed. Therefore, it displayed a concave in the center of the geometric position, with a relatively large curvature in the peak of the contour and a smaller amplitude. 

Then, in Figure 7b, as the red line was closer to the boundary in different directions, the corresponding contour showed a gentler “∪” in the peak and a larger amplitude. 

The middle points of the two red lines were taken as an example; in one-dimension heat transfer, the two points were assumed to be at the same depth and therefore have the same temperature value, while in three-dimension heat transfer, there was a noticeable temperature difference between the two points in the peak of the contour, as for other points at the same depth. Obviously, the former had radical shortcomings, and the latter could better realize the prediction of the internal temperature. In addition, the phenomenon revealed the fact that the temperature disturbance was determined by multi-dimensional heat transfer. In contrast to one-dimension heat transfer, the closer points are to the different boundaries, the more frequently temperature alternates, explaining how this model improved the precision.

## 4. Application of the Model

In order to investigate the application of the model in the prediction of concrete temperature under different conditions, the simulation of concrete specimens was conducted, and the environment parameters of Nanjing (China) were given as the boundary conditions.

### 4.1. Representative Data Selection

**Figure 8:**
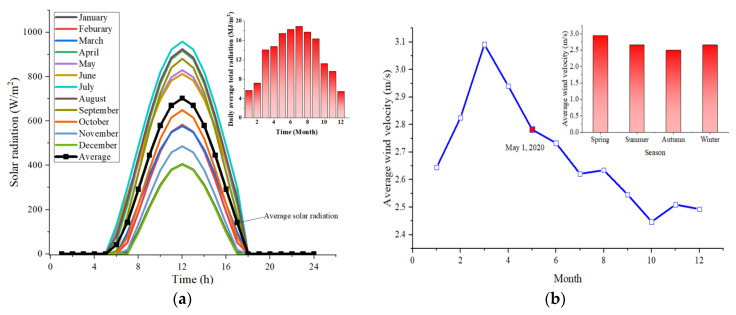

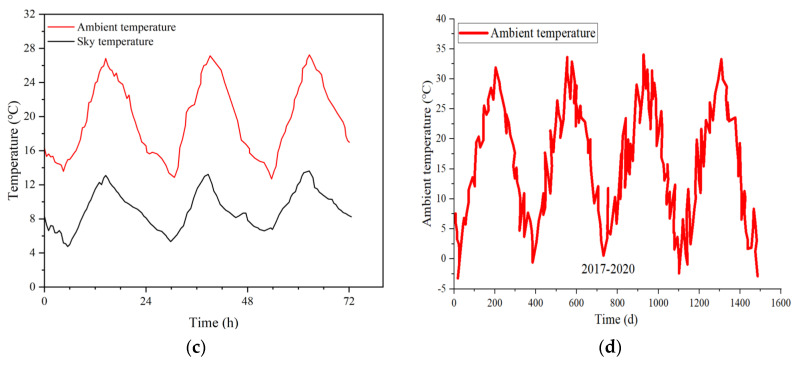
Measured environment parameters used for the numerical simulation. (**a**) Solar radiation (2020); (**b**) Wind velocity (2020); (**c**) Ambient temperature (1–3 May 2020); (**d**) Annual temperature curve (2017–2020).

The primary environment parameters of Nanjing derived from the China Meteorological Administration (CMA) [42], such as solar radiation, wind velocity, and ambient temperature, are indicated in Figure 8. 

The climate conditions of Nanjing varied over time. In order to reflect the general situation and meet the requirements of authenticity and representativeness, 1−3 May 2020 was set as the representative date, for which the climate data were consequently incorporated into the model.

Specifically, Figure 8a shows the distribution of solar radiation within a day in different months and the daily average total radiation from 12 months. The black line refers to that of the representative day. Figure 8b depicts the mean wind velocity of 12 months and four seasons, where the red plot is the value of the representative day. Figure 8c describes the ambient temperature and sky temperature curve of the representative day, derived from the median value of Figure 8d.

### 4.2. Finite Element Model

**Figure 9:**
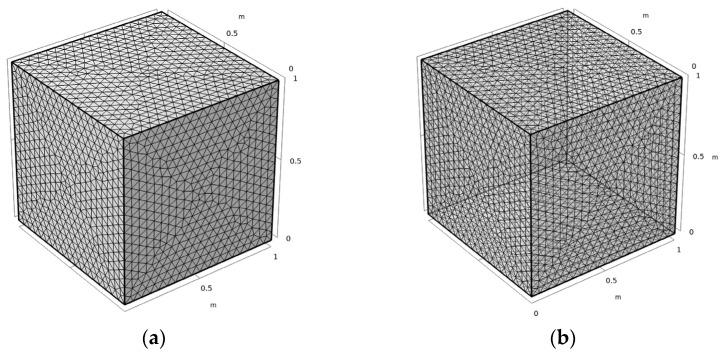
The finite element mesh model for the simulation. (**a**) Stereogram; (**b**) Perspective.

Similar to the FEM model mentioned above, a concrete specimen with dimensions of 1000 × 1000 × 1000 mm^3^ was used. The entire flat surfaces were meshed into 4692 triangular boundary elements and the internal domain into 100,553 tetrahedron domain elements, as shown in Figure 9. The initial temperature was designated as 15 °C. The implementation of the environmental conditions was the same as that mentioned in Chapter 3.2. The bottom surface temperature was set to be 20 °C, based on the daily average land surface temperature in May [40]. The concrete mix proportions were the same as that in Table 1.

### 4.3. Results of the Simulation

In order to study the relationship between temperature response at different depths, seven points along the diagonal line from outside to inside (Node1-7 stand for the points (0.5, 0.5, 0.5), (0.6, 0.6, 0.6), (0.7, 0.7, 0.7), (0.8, 0.8, 0.8), (0.9, 0.9, 0.9), (0.99, 0.99, 0.99), and (1, 1, 1) in a 3D coordinate system) were selected. 

**Figure 10:**
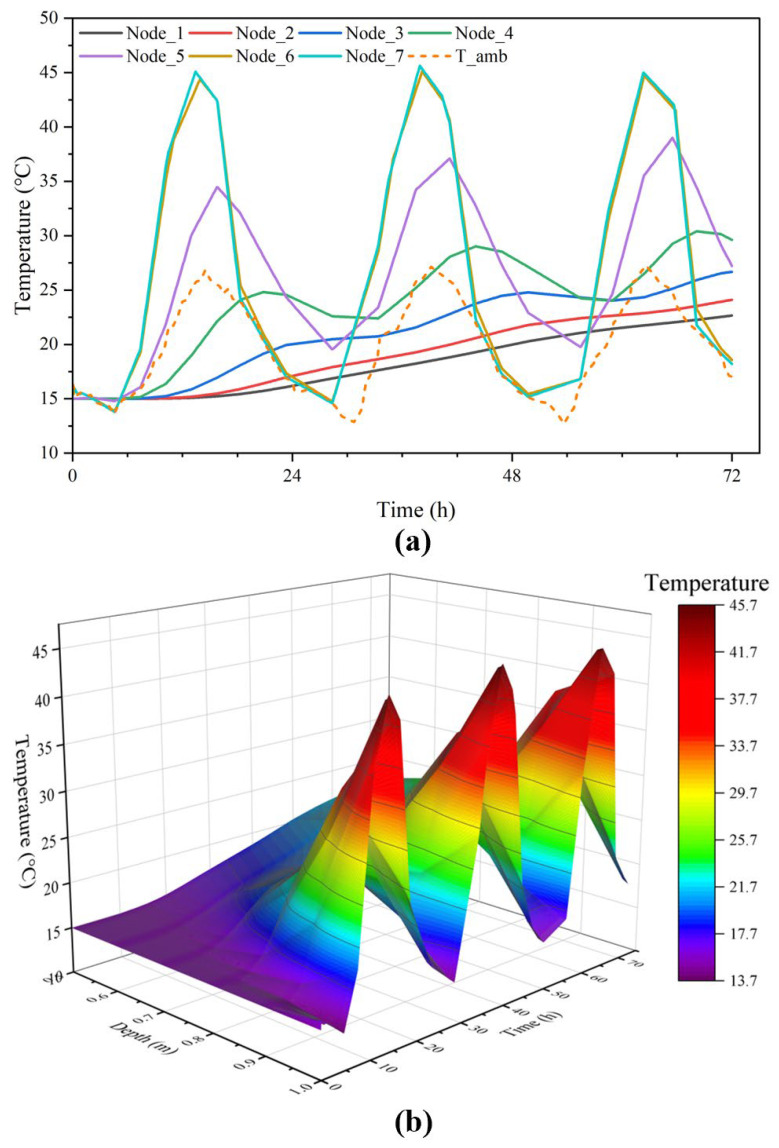
Time-depended temperature distribution in different depth. (**a**) Temperature response of all points from Node_1 to Node_7; (**b**) Temperature response contour of the line where all points are located.

Figure 10a shows temperature response of all points from Node_1 to Node_7 inside the concrete specimen, and Figure 10b depicts temperature response contour of the line where all points are located.

It is apparent from Figure 10a,b that the fluctuation of temperature became smaller with the increases in depth. The following results are drawn:

1. The concrete specimen exposed to the fluctuating ambient temperature from an isothermal state generated significant temperature differences from outside to inside. Each day, the surface had a temperature fluctuation of almost 30 °C, six times that of the middle. From 08:00 a.m. to 13:00 p.m., ambient temperature and solar radiation rose sharply, temperatures of the outer parts increased quicker than the inner part, and then the maximum temperature difference reached 25 °C at 15:00 p.m.
2. The hysteresis effect intensified as the depth increased. Figure 10a shows gigantic inside and outside temperature differences over time. Figure 10b shows the isotherms bend with the increase of depth.
3. In the process of temperature response, the temperature values not only increased or decreased from outside to inside with time, but also changed irregularly. For example, before 17:00 p.m., temperature values basically increased from inside to outside. However, after that, the values first increased and then decreased. Therefore, it could be assumed that thermal expansion and contraction occured between different layers. Based on this study, the prediction model of temperature stress distribution remained to be investigated.

### 4.4. Sensitivity Evaluation

After the model was verified, a sensitivity analysis was conducted to evaluate the influences of factors on temperature distribution in concrete, such as convection, solar radiation, and material properties. 

The conditions of 1–3 May 2020 were taken as a reference; when one factor was evaluated, the others remained the reference conditions to draw the impact of a single variable on the temperature distribution. 

#### 4.4.1. Effect of Convection

To consider the influence of atmospheric convection, wind velocities at 0 m/s, 5 m/s, and 10 m/s were introduced, with an average wind velocity of 2.7 m/s being taken as the reference case. Three points in different depths were considered and could be expressed as (1, 1, 1), (0.9, 0.9, 0.9), and (0.5, 0.5, 0.5) in the 3D coordinate system. 

**Figure 11:**
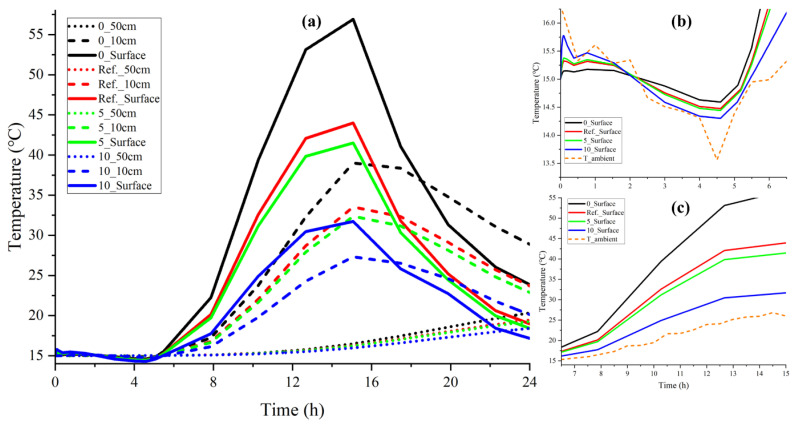
Impact of convection on the temperature distribution (0 m/s set as blank control group): (**a**) three depths: 0 m, 10 cm, and 50 cm; (**b**) surface temperature from 00:00−06:00 a.m.; (**c**) surface temperature from 07:00 a.m.–15:00 p.m.

As depicted in Figure 11a, there were significant differences between each surface temperature curve when changing the wind velocity. For example, the maximum surface temperature of the blank control group was twice greater than that of the 10 m/s. However, due to convection mainly working on the surface and the influence of heat transfer in the medium, the temperature fluctuation decreased with depth, regardless of the different wind speeds. 

It can be derived from Figure 11b,c that higher wind speed accompanied with a better cooling effect made the surface temperature closer to the ambient temperature, because the greater the wind speed, the greater the frequency of temperature exchange, and the more it achieved heat equilibrium with the ambient temperature. While the small wind speed caused heat accumulation, especially with no wind, the heat equilibrium between the surface and the environment depended mainly on the heat conduction induced by the temperature gradient. This explains why the temperature rise of this group was relatively quicker.

In conclusion, convection had a noticeable impact on heat transfer by promoting the heat exchange rate. The model was sensitive to wind speed change and depicted the cooling effect well.

#### 4.4.2. Effect of Solar Radiation

As sky radiation is related to the ambient temperature and sky temperature, solar radiation was taken into consideration in the sensitivity evaluation. 

The solar radiation of weak intensity (December), moderate intensity (reference case), strong intensity (July), and zero intensity (0 W/m^2^) were taken into consideration. As mentioned before, three points in different depths were investigated. 

**Figure 12:**
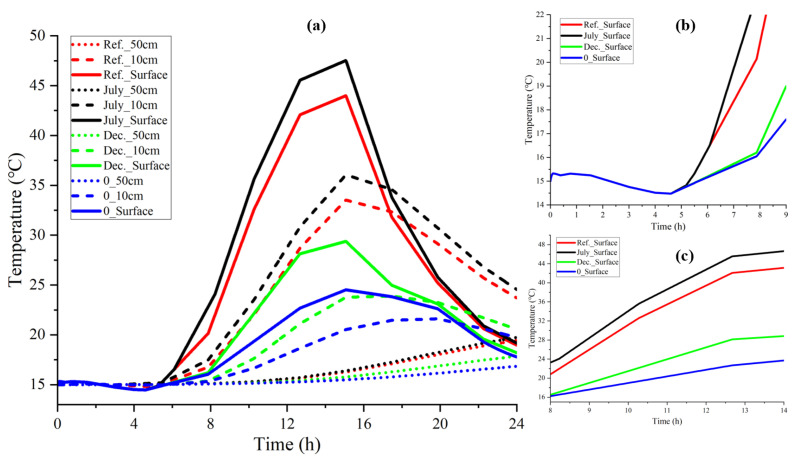
Impact of solar radiation on the temperature distribution (0 W/m^2^ set as blank control group) (**a**) Three depths: 0 m, 10 cm, 50 cm; (**b**) Surface Temperature from 0–9 o’clock; (**c**) Surface Temperature from 8–14 o’clock.

It can be seen from Figure 12a that solar radiation had a tremendous impact on heat transfer.

Under different solar radiation intensities, the amplitude of the surface temperature curve and the internal and external temperature difference increased with the solar radiation intensity. For example, the surface temperature fluctuation amplitude in July was almost three times that of the blank control group. Nevertheless, the differences declined with depth increases due to heat transfer in the medium. At a depth of 50 cm, the temperature difference between each curve was less than 5 °C. When exposed to a specific solar intensity, surface temperature changed quickly, but the fluctuation gradually declined with the increase of depth.

Figure 12b shows that the surface temperature was sensitive to solar radiation; once it emerged, the temperature curves diverged quickly. As shown in Figure 12c, more vigorous solar radiation intensity produces a more significant surface temperature increment, because the greater solar radiation intensity inputs more energy, produces heat accumulation, and consequently results in the surface temperature increasing quickly. However, the differences between each curve decreased with depths.

In conclusion, the solar radiation has a remarkable impact on heat transfer by increasing the total heat input. The model was tested to be sensitive to solar radiation.

#### 4.4.3. Effect of Material Properties

The thermodynamic performance of concrete depends significantly on several physical properties, such as the density, specific heat capacity, and thermal conductivity. 

**Figure 13:**
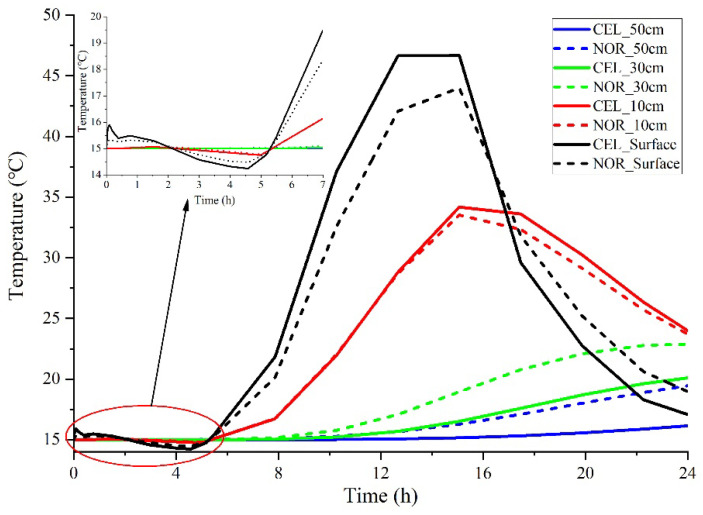
Impact of material properties on temperature distribution. CEL: cellular concrete; NOR: normal concrete).

Regular concrete (ordinary Poland cement), wildly used as a primary structural material, was proven to be applicable in the prediction model. In addition, cellular concrete was widely used as an essential component in building thermal insulation materials. The physical properties of regular concrete and cellular concrete are listed in Table 3, and the mixture proportion of regular concrete is shown in Table 1. The impact of material properties on temperature distribution is shown in Figure 13.

It can be seen from Figure 13 that significant differences existed in the sensitivity to ambient temperature between regular concrete and cellular concrete. 

First, the variation amplitude of the surface temperature of cellular concrete was greater than that of regular concrete. Specifically, when the environmental temperature decreased, the surface temperature of cellular concrete was lower than that of regular concrete; when environmental temperature increased, the surface temperature of cellular concrete was greater because cellular concrete had a lower specific heat capacity than regular concrete.

Then, differing from the surface temperature, there was little difference between temperature curves at a depth of 10 cm. The surface temperature difference was offset because there were differences in internal thermal conductivity between cellular and regular concrete. Heat transfer coefficient plays the dominant role in internal temperature distribution, which was further confirmed by the temperature difference between cellular and regular concrete at deeper depths. For example, at depths of 30 cm and 50 cm, the variation amplitude of the temperature curve of cellular concrete was smaller than that of regular concrete. Based on the above analysis, 10 cm can roughly serve as the dividing line for where the heat preservation effect of cellular concrete emerges.

Therefore, the material properties have a significant effect on the model. When shifting the different material properties of cellular concrete or other thermal insulation materials, our model can predict the depth where the heat preservation effect emerges compared to regular concrete.

## 5. Conclusions

A heat transfer model coupled with convection, solar radiation, and fluctuating ambient temperature was established based on theoretical analysis and finite element calculation, in order to predict the temperature distribution of a concrete structure under a natural environment. Comparison and sensitivity analysis between the predicted internal temperature of concrete and the actual-measured data were also conducted, from which the following conclusions could be drawn:

1. Three-dimensional transient heat transfer model coupling with convection, solar radiation, and ambient temperature was developed to predict the temperature distribution inside the concrete. The comparison between the predicted results and the real-measured results showed good consistency.
2. Sensitivity evaluation revealed that the convection, solar radiation, and material properties had an evident influence on heat transfer and temperature distribution in concrete: Greater wind speed promoted the heat exchange rate between the surface and environment, making the surface temperature near to the ambient temperature; solar radiation increased the total heat input, resulting in rapid temperature rise; the specific heat capacity and heat transfer coefficient had significant impacts on the surface temperature distribution and internal temperature distribution, respectively.
3. The three-dimensional heat transfer model discovered a distinct temperature difference between different points of the same depth, proving the temperature prediction results of the three-dimensional model was relatively more accurate than the one-dimensional model.
4. Temperature inside the concrete tended to be retarded to the fluctuating ambient temperature, with the depth in the vertical direction being increased by 50 cm, the variation amplitude being decreased by more than 90%.
5. The internal temperature was affected by the ambient temperature; sometimes, it rose from inside to outside, but sometimes it ascended first and then declined due to different response speeds. Thus, thermal expansion and cold contraction existed between different layers at different times, in which the problem of stress field prediction needs solving.

## Figures and Tables

**Table 1:** Concrete mix proportion [24].

	Cement	Water	Fine Aggregate	Coarse Aggregate	W/C
Mixture(kg/m^3^)	297	178	861	950	0.6

**Table 2:** Parameters of the concrete and boundary conditions.

Parameters	Equations	Features	Refs.
Thermal conductivity	${k=k}_{m}\left(2M-M^{2}\right)\frac{k_{m}k_{a}{(1-M)}^{2}}{k_{a}{M+k}_{m}\left(1-M\right)}$ $M=1-{(1-p)}^{\frac{1}{3}}$ $E=\frac{{\mathrm{nk}}_{a}}{\left(n-1\right)k_{s}{+k}_{f}}$	k stands for thermal conductivity and the subscripts m and a represent mortar and aggregate, respectively, and p is the volume of mortar in each unit volume of concrete. $k_{s}$ refers to the thermal conductivity of the continuous phase, $k_{f}$ to the disperse phase, and n to the geometrical distribution function of different phases.	[40]
Sky temperature	$T_{\mathrm{sky}}{=T}_{a}{\left[\begin{array}{c} 0.711+{0.0056T}_{\mathrm{dp}\;}+{0.000073T}_{\mathrm{dp}}^{2}\\ +0.013\cos\left(15\;t\right) \end{array}\right]}^{\frac{1}{4}}$	$T_{\mathrm{dp}}$ refers to dew point temperature.	[22]
Density	2300 kg/m^3^		
Specific Heat	$C_{\mathrm{conc}}^{p}{=C}_{\mathrm{water}}^{p}m_{\mathrm{water}}{+C}_{\mathrm{cem}}^{p}m_{\mathrm{cem}}{+C}_{\mathrm{FA}}^{p}m_{\mathrm{FA}}{+C}_{\mathrm{sand}}^{p}{+m}_{\mathrm{aggr}}C_{\mathrm{aggr}}^{p}$	$c^{p}$ is specific heat capacity; m refers to mass fraction; and the subscripts conc, water, cem, FA, sand, and aggr represent concrete, water, cement, fly ash, sand, and aggregate, respectively.	[41]

**Table 3:** Material properties of cellular concrete.

Materials	Specific Heat Capacity	Density	Thermal Conductivity
Cellular concrete	850 J/(kg·K)	600 kg/m^3^	0.14 W/(m·K)
Regular concrete	970 J/(kg·K)	2300 kg/m^3^	1.51 W/(m·K)

## Data Availability

Not applicable.
